# Implementation Evaluation of HUGS/Abrazos During the COVID-19 Pandemic: A Program to Foster Resiliency in Pregnancy and Early Childhood

**DOI:** 10.3389/fpubh.2022.862388

**Published:** 2022-05-20

**Authors:** Meisui Liu, Meg Simione, Meghan E. Perkins, Sarah N. Price, Mandy Luo, William Lopez, Viktoria M. Catalan, Szu-Yu Tina Chen, Carlos Torres, Gracia M. Kwete, Molly Seigel, Andrea G. Edlow, Maria Yolanda Parra, Mary Lyons Hunter, Alexy Arauz Boudreau, Elsie M. Taveras

**Affiliations:** ^1^Division of General Pediatrics, Department of Pediatrics, Massachusetts General Hospital for Children, Boston, MA, United States; ^2^Department of Pediatrics, Harvard Medical School, Boston, MA, United States; ^3^Harvard Medical School, Boston, MA, United States; ^4^MGH Chelsea HealthCare Center, Chelsea, MA, United States; ^5^MGH Revere HealthCare Center, Revere, MA, United States; ^6^Department of Obstetrics and Gynecology, Massachusetts General Hospital, Boston, MA, United States; ^7^Kraft Center for Community Health, Massachusetts General Hospital, Boston, MA, United States; ^8^Department of Nutrition, Harvard T.H. Chan School of Public Health, Boston, MA, United States

**Keywords:** toxic stress, patient navigation, resilience, RE-AIM, COVID-19 pandemic, early childhood

## Abstract

Early life adversity can significantly impact child development and health outcomes throughout the life course. With the COVID-19 pandemic exacerbating preexisting and introducing new sources of toxic stress, social programs that foster resilience are more necessary now than ever. The Helping Us Grow Stronger (HUGS/Abrazos) program fills a crucial need for protective buffers during the COVID-19 pandemic, which has escalated toxic stressors affecting pregnant women and families with young children. HUGS/Abrazos combines patient navigation, behavioral health support, and innovative tools to ameliorate these heightened toxic stressors. We used a mixed-methods approach, guided by the Reach, Effectiveness, Adoption, Implementation, and Maintenance (RE-AIM) framework, to evaluate the implementation of the HUGS/Abrazos program at Massachusetts General Hospital from 6/30/2020–8/31/2021. Results of the quality improvement evaluation revealed that the program was widely adopted across the hospital and 392 unique families were referred to the program. The referred patients were representative of the communities in Massachusetts disproportionately affected by the COVID-19 pandemic. Furthermore, 79% of referred patients followed up with the initial referral, with sustained high participation rates throughout the program course; and they were provided with an average of four community resource referrals. Adoption and implementation of the key components in HUGS/Abrazos were found to be appropriate and acceptable. Furthermore, the implemented program remained consistent to the original design. Overall, HUGS/Abrazos was well adopted as an emergency relief program with strong post-COVID-19 applicability to ameliorate continuing toxic stressors while decreasing burden on the health system.

## Introduction

Early life adversity, defined as recurrent stressful events that occur during sensitive periods of development, can have profound impact on child development and health outcomes throughout the life course (1–4). Pregnancy and the early childhood years are examples of critical periods of development during which the parent-child dyad is more vulnerable to toxic stressors (5). Adversity during these critical periods of development influences neurodevelopmental processes at the cellular level (6, 7), disrupts normal immunoregulatory scaffolding (8), and results in cumulatively increased risk for disease in adulthood (2, 8, 9). Furthermore, the early life environment can exert intergenerational impact on risk for chronic disease throughout the life course via epigenetic mechanisms (10). Reassuringly, protective buffers can curb the negative impact of toxic stress and build resilience among children and families experiencing adversity (11–14). Therefore, social programs that foster resilience are necessary.

The COVID-19 pandemic exacerbated preexisting and introduced new sources of toxic stress for families with young children. Specifically, the newfound challenges of at-home parenting (15), financial insecurity (16, 17), racial disparities in health outcomes (18–20), and behavioral health burdens have all escalated to critical levels (21–23). This is especially true for marginalized communities such as racially and ethnically diverse populations, immigrants, and families in poverty (18, 19, 24–26). The Helping Us Grow Stronger (HUGS/Abrazos) program fills a crucial need for protective buffers during the COVID-19 pandemic, which has escalated toxic stressors affecting pregnant women and families with young children. The multimodal strategy utilized by HUGS/Abrazos to support patients from communities hardest hit by the COVID-19 pandemic has been previously described (27). Specifically, HUGS/Abrazos supports patients served by Massachusetts General Hospital (MGH), including MGH community health centers in Chelsea and Revere, two communities most severely impacted by the COVID-19 pandemic in Massachusetts.

Given the quickly evolving public health crisis, analytical methods that can assess public health interventions without delaying implementation are crucial. RE-AIM – Reach, Effectiveness, Adoption, Implementation, and Maintenance – provides an evaluation framework that assesses the delivery of public health interventions while bridging the gap between practice and research (28–30). The RE-AIM framework has been especially helpful when used to inform adaptations and dissemination of interventions in low resource settings (31–33). We used a mixed-methods approach, guided by the RE-AIM framework, to evaluate the implementation of the HUGS/Abrazos program to inform future program adaptations, dissemination, and sustainability.

## Methods

### Overview of HUGS/Abrazos Program

The design and implementation of the HUGS/Abrazos program has been previously described (27). HUGS/Abrazos aimed to (1) use targeted patient navigation to address unmet health-related social needs; (2) provide short-term, immediate behavioral health support; and (3) create cross-systems linkages among community partners using centralized resource repository and an integrated referral system. The targeted patient population included communities in and surrounding Boston, MA that were heavily impacted by the COVID-19 pandemic. These communities had higher number of immigrants, families living in poverty, and residents of racially and ethnically minoritized groups compared to state average. Eligibility criteria included (1) pregnant women and families with children under 6 years old; (2) demonstration of an unmet socioeconomic or behavioral health need; and (3) had an established provider within MGH system. In a cross-departmental collaboration, providers in Pediatrics, Obstetrics & Gynecology (OB/Gyn), Family Medicine, and other specialties made initial referrals to HUGS/Abrazos. Referrals were triaged and assigned to a community health worker (CHW), who navigated patients toward community resources, or to the behavioral health team, who provided behavioral health support, or to both. During the first encounter, a screening questionnaire was used to assess for specific unmet socioeconomic or behavioral health needs. Referred patients connected with the CHW for up to three touchpoints, and with the behavioral health team for up to four touchpoints. We utilized a centralized resource repository in Aunt Bertha and the Integrated Referral and Intake system (IRIS) to streamline communication and workflow. All patients received a care package that included a $50 gift card for groceries, age-appropriate activity kits, and supplies to encourage healthy practices during the pandemic. At the conclusion of the program, patients were referred to long term services if necessary and available. HUGS/Abrazos conception and design began in April 2020, and program launch occurred in July 2020. Evaluation of implementation included patients referred to the program between 6/30/2020 and 8/31/2021. The Mass General Brigham Institutional Review Board determined the evaluation of the HUGS/Abrazos program to be local program evaluation intended for quality improvement purposes and did not require Institutional Review Board oversight.

### Overview of Mixed Methods Evaluation Using the RE-AIM Framework

We used a mixed methods approach, guided by the RE-AIM framework, to evaluate the implementation and delivery of HUGS/Abrazos (30). Quantitative data related to reach, effectiveness, and adoption (R,E,A) were obtained from the electronic health record (EHR) and administrative data. Qualitative data related to implementation and maintenance (I, M) were obtained through focus group sessions, which subsequently underwent rapid qualitative analysis described below. See Table 1 for the specific measures used to assess each domain of the RE-AIM framework.

**Table 1:** Evaluation of the HUGS/Abrazos program using the RE-AIM framework.

**RE-AIM Component**	**Measure**	**Data source**
Reach	Total number of patients seen in practices referring to HUGS	Electronic Health Record (EHR)
	Number of unique referrals made to HUGS	
	Number of families who completed ≥ 1 touchpoint	
	Socio-demographics of referred patients	
Effectiveness	Number of touchpoints with community health, behavioral health, and community health + behavioral health combined	Electronic Health Record (EHR)
	Average number of referrals provided to community resources	
	Reason for referrals to community resources	
Adoption	Characteristics of practices referring to HUGS	Administrative and EHR data
	Characteristics of providers making referrals to HUGS	
Implementation	Appropriateness of HUGS	Qualitative focus groups with community health, behavioral health, and physician champions
	Acceptability of HUGS	
	Penetration of HUGS	
	Fidelity to the program and adaptations made	
Maintenance	Sustainability of HUGS	Qualitative focus groups with community health, behavioral health, and physician champions

*RE-AIM, Reach, Effectiveness, Adoption, Implementation, Maintenance*.

### Quantitative Evaluation Methods

To evaluate adoption, defined as the representativeness of settings that implement a new program (29), we utilized the EHR and administrative data to determine the characteristics of practices and providers who made referrals to HUGS/Abrazos.

To evaluate reach, defined as the participation rate and characteristics of the program-eligible population (29), we utilized the EHR to determine the number of patients seen in practices referring to HUGS/Abrazos and the number of unique referrals made to HUGS/Abrazos. Due to the possibility in which multiple referrals were made for the same family or individual for different reasons, we tracked the number of unique families referred. After we identified families, we then selected the first referral made and used that information when reporting. When possible, we linked child and parent data. Of the referrals made, we determined the number of families who completed at least one touchpoint during their HUGS/Abrazos participation. We summarized the socio-demographics of referred patients, which included parent age, child age, sex, race and ethnicity, language, birth country, insurance status, education level, marital status, and employment status, and stratified by those who completed touchpoints and those who did not.

To evaluate the effectiveness, defined as the impact of the program for the participating population (29), we utilized the EHR to determine the number touchpoints patients completed with the CHW, the behavioral health team, or both. Additionally, we determined the reasons for which referrals to community resources were made and the average number of referrals provided to patients for community resources.

We calculated descriptive statistics for the number of referred patients, unique referrals to HUGS/Abrazos, touchpoints completed, and the referrals to community resources. We performed statistical analyses using RStudio 1.4.1717 (R Core Team) (34).

### Qualitative Evaluation Methods

We designed and facilitated three focus group sessions with stakeholder groups to elucidate their perspectives on the implementation and maintenance of HUGS/Abrazos. The first focus group session involved all the CHWs who provided patient navigation of community resources appropriate to each patient's health related social needs (*n* = 6). The second focus group session involved all members of the behavioral health team, who provided stress reduction strategies, mindfulness techniques, and cognitive behavioral therapy to alleviate acute behavioral health needs (*n* = 3). Finally, the third focus group session involved primary care physicians (PCP), who were part of the program's initial conception and design team and served as clinician champions heralding the program's launch (*n* = 2). The focus group interview guide was developed according to the sustainability-enhanced RE-AIM framework with questions tailored to evaluate implementation outcomes, which included appropriateness, acceptability, penetration, fidelity to program design, and sustainability (30, 35, 36).

We used rapid qualitative analysis methods that have been successfully used in prior studies to inform implementation when results are needed in a timely manner (37, 38). We recorded and transcribed the focus group sessions for rapid qualitative analysis to determine key findings related to implementation and maintenance of HUGS/Abrazos. First, the evaluation team created a summary table that outlined (1) each implementation outcome with its associated focus group questions; (2) key findings; and (3) related exemplar quotes in the transcript. Next, the analytic team extracted data from one focus group transcript to populate the summary table. The evaluation team then reviewed and modified the summary table based on the initial analysis of one transcript. The analytic team subsequently extracted data from remaining focus group transcripts to populate the newly modified summary tables. A second review of the summary tables by the evaluation team was performed to ensure accuracy and consistency in data extraction. Finally, the summary tables were used to populate a matrix in Microsoft Excel to identify themes and subthemes consistent across stakeholder groups. The evaluation team reviewed and discussed the matrix to finalize the themes. The conception and design team of HUGS/Abrazos performed a final review of the identified themes and subthemes.

## Results

### Adoption

A multidisciplinary cohort of providers from 31 different MGH site specific departments, grouped into 12 overall department categories, referred patients to the HUGS/Abrazos program. Most referrals originated from Pediatrics/Adolescent Health (38.0%), Obstetrics (31.1%), Family Medicine (11.7%), and Behavioral Health/Psychiatry (6.6%) which correlates well with the HUGS/Abrazos intended patient population of pregnant women and families with children under 6 years old. Remaining referrals to HUGS/Abrazos originated from department categories that include but are not limited to emergency medicine, care coordination, social services, and more. Referring providers included physicians, midwives, psychologists, social workers, nurse practitioners, and others.

The MGH HealthCare Centers in Chelsea and Revere were the most common referral sites, making up 42.6 and 23.5%, respectively, of parent referrals and 62.4 and 22.0%, respectively, of child referrals. This correlates well with Chelsea and Revere being the primary sites of HUGS/Abrazos' initial design and implementation. However, 33.9 and 15.6% for parent and child referrals, respectively, originated from MGH main hospital and other MGH affiliated community-based health centers, suggesting successful widespread multi-site adoption of HUGS/Abrazos in the MGH hospital system.

### Reach

A total of 6,267 women and 8,055 children under 6 years old were seen for obstetric and pediatric care, respectively, at practices participating in HUGS/Abrazos since its implementation. A total of 551 referrals were made for HUGS/Abrazos, and of this, 392 referrals were made for unique families during the evaluation period (ex. one family may be referred for both food insecurity and baby supplies). The racial and ethnic demographics of referred patients were comparable to the racial and ethnic demographics of the communities in which we targeted our outreach efforts. For example, the racial and ethnic demographics of parents in the HUGS/Abrazos program included 64.2, 22.9, and 8.5% of Hispanic, non-Hispanic Whites, and non-Hispanic Blacks in comparison to 67.0, 20.6, and 5.4% of the same racial and ethnic groups, respectively, in Chelsea, MA (39). A greater percentage of parents referred to HUGS/Abrazos were foreign-born compared to the percentage of foreign-born residents in Chelsea, MA (67.6 vs. 45.4%) (39). Meanwhile, most children referred to HUGS/Abrazos were born in the United States (93.4%). A majority of referred patients, 41.3% for parent referrals and 61.1% of parents of referred children, spoke a language other than English as their primary language. We also obtained the demographic data for patients who were referred to HUGS/Abrazos but did not participate in any touchpoints for a full scope of our reach. Please see Table 2 for detailed demographics data of the HUGS/Abrazos patient population.

**Table 2:** Characteristics of families referred to the HUGS/Abrazos program (*N* = 392).

	**All referred parents**	**Parents who engaged in HUGS**	**Parents who did not engage in HUGS**	**All referred children**	**Children whose parents engaged in HUGS**	**Children whose parents did not engage in HUGS**
	***N*** **=** **206**	***N*** **=** **164**	***N*** **=** **42**	***N*** **=** **186**	***N*** **=** **146**	***N*** **=** **40**
**Age at referral**						
Mean (SD)	29.5 (6.7)	29.7 (6.8)	28.6 (6.2)	1.8 (1.8)	1.9 (1.8)	1.6 (1.8)
Range	(16.4, 56.0)	(16.4, 56.0)	(18.7, 42.9)	(0.0, 7.0)	(0.0, 6.6)	(0.0, 7.0)
**Age categories of children at initial referral**						
0–5.9 mo.	N/A	N/A	N/A	55 (29.6)	42 (28.8)	13 (32.5)
6.0–11.9 mo.	N/A	N/A	N/A	28 (15.1)	21 (14.4)	7 (17.5)
1.0–1.9 yrs.	N/A	N/A	N/A	30 (16.1)	21 (14.4)	9 (22.5)
2.0–3.9 yrs.	N/A	N/A	N/A	41 (22.0)	35 (24.0)	6 (15.0)
≥ 4.0 yrs.	N/A	N/A	N/A	32 (17.2)	27 (18.5)	5 (12.5)
**Sex**, ***n*** **(%)**						
Male	2 (1.0)	2 (1.2)	0 (0)	90 (48.4)	69 (47.3)	21 (52.5)
Female	204 (99.0)	162 (98.8)	42 (100.0)	96 (51.6)	77 (52.7)	19 (47.5)
**Race/Ethnicity**, ***n*** **(%)**		***N*** **=** **201**		***N*** **=** **135**		
White	46 (22.9)	28 (17.5)	18 (43.9)	8 (5.9)	5 (4.7)	3 (10.3)
Hispanic or Latino	129 (64.2)	114 (71.2)	15 (36.6)	108 (80.0)	87 (82.1)	21 (72.4)
Black or African American	17 (8.5)	12 (7.5)	5 (12.2)	12 (8.9)	9 (8.5)	3 (10.3)
Asian or Multiracial	9 (4.5)	6 (3.8)	3 (7.3)	7 (5.2)	5 (4.7)	2 (6.9)
**Language**, ***n*** **(%)**		***N*** **=** **206**		***N*** **=** **185**		
English	121 (58.7)	89 (54.3)	32 (76.2)	72 (38.9)	49 (33.8)	23 (57.5)
Spanish	75 (36.4)	69 (42.1)	6 (14.3)	100 (54.1)	85 (58.6)	15 (37.5)
Other	10 (4.9)	6 (3.7)	4 (9.5)	13 (7.0)	11 (7.6)	2 (5.0)
**Birth Country**, ***n*** **(%)**		***N*** **=** **176**		***N*** **=** **182**		
Foreign Born	119 (67.6)	103 (73.0)	16 (45.7)	12 (6.6)	12 (8.4)	0 (0)
**Insurance**, ***n*** **(%)**			
Public	163 (79.1)	132 (80.5)	31 (73.8)	172 (92.5)	136 (93.2)	36 (90.0)
Private	43 (20.9)	32 (19.5)	11 (26.2)	14 (7.5)	10 (6.8)	4 (10.0)
**Education**, ***n*** **(%)**		***N*** **=** **199**				
Some high school or less	59 (29.6)	52 (32.9)	7 (17.1)	N/A	N/A	N/A
High school graduate	73 (36.7)	59 (37.3)	14 (34.1)	N/A	N/A	N/A
More than high school or other	67 (33.7)	47 (29.7)	20 (48.8)	N/A	N/A	N/A
**Marital Status**, ***n*** **(%)**		***N*** **=** **204**				
Unmarried	123 (60.3)	100 (61.7)	23 (54.8)	N/A	N/A	N/A
**Employment**, ***n*** **(%)**		***N*** **=** **198**				
Unemployed	122 (61.6)	99 (62.7)	23 (57.5)	N/A	N/A	N/A

### Effectiveness

**Figure 1:**
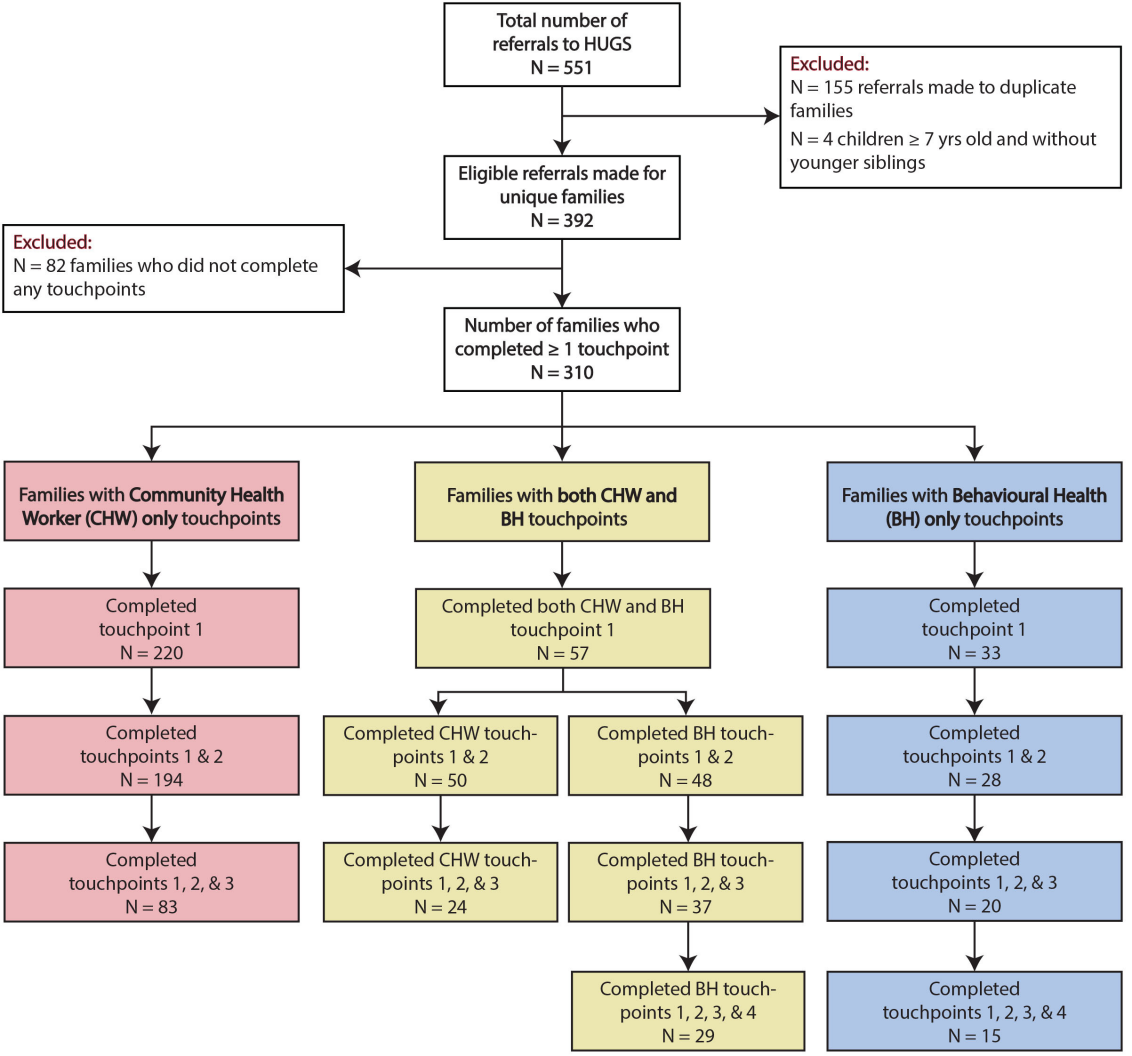
Flowchart of referrals to the HUGS/Abrazos Program (6/30/2020–8/31/2021). Patients can complete up to three touchpoints with the community health workers or up to four touchpoints with the behavioral health team.

A total of 392 unique families were referred to HUGS/Abrazos (see Figure 1). Of these families, 310 or 79% of them completed at least one touchpoint with either a CHW or the behavioral health team. Although we did not collect data on reasons for non-participation, possible explanations may include newfound access to another resource, inability to participate due to time constraints and other stressors, access to technology for virtual visits, language and cultural barriers despite availability of interpreters, fear of social stigma, and anxiety around immigration status. Participating families maintained longitudinal relationships through multiple touchpoints with either the CHW, the behavioral health team, or both. Of the 220 families who completed CHW only services, 194 completed at least two touchpoints, and 83 completed three touchpoints. Of the 33 families who completed behavioral health only services, 28 completed at least two touchpoints, 20 completed at least three touchpoints, and 15 completed the maximum four touchpoints. A total of 57 families received both CHW and behavioral health services. Participation rates in either CHW or behavioral health team touchpoints for these families trended similarly to the data for CHW only and behavioral health only families. Families who engaged in patient navigation services with the CHW were referred to an average number of 4.4 community resources and had an average of 3.71 reasons for these referrals (Supplementary Table 1). The most common reasons for community resource referrals included but are not limited to infant supplies (60.2%), food security (52.3%), and support with housing and related legal issues (48.5%) (Supplementary Table 1).

### Implementation and Maintenance

Table 3 summarizes the themes and subthemes related to the implementation and maintenance of HUGS/Abrazos, as well as exemplar quotes, that emerged from rapid qualitative analysis of focus group sessions with CHWs, the behavioral health team, and PCP champions. All focus group participants were females except for two male participants. Professional roles included: CHW, social worker, psychologist, and pediatricians. When assessing appropriateness, or the perceived fit of the program for its intended audience and setting (35), three key subthemes emerged: (1) HUGS/Abrazos successfully targeted its patient outreach toward peripartum women and families with unmet socioeconomic and behavioral health needs, (2) provided them with emergency relief of the most acute issues, and (3) did so in a manner that maximized equity and accessibility. When assessing acceptability, or the perception among stakeholders that the program is agreeable (35), HUGS/Abrazos was perceived as a successful multidisciplinary collaboration that simultaneously reduced the burden on an already overwhelmed behavioral health system and led to positive impacts on patients' lives. When assessing fidelity, or the degree to which the program was implemented as it was designed (35), most stakeholders found that the core components of HUGS/Abrazos – patient navigation, acute behavioral health support, and utilization of a centralized resource repository and integrated referral system – were implemented with high fidelity though flexibility was necessary during individual interactions to fulfill differing needs. In terms of program penetration (35), or the degree to which the program has integrated into the existing infrastructure, HUGS/Abrazos successfully closed the gap among previously siloed resources and care teams to forge new coalitions and relationships within the hospital system and with community partners. From the perspective of the HUGS/Abrazos PCP champions, behavioral health teams, and CHWs, implementation barriers and program limitations included technological challenges in setting of virtual visits, lack of interpreter services at partner organizations, and sensitivity of conversations around immigration status, all of which may directly influence the effectiveness of the program for participants. Finally, stakeholders believe there is value in maintaining HUGS/Abrazos beyond the pandemic as the need for social programs that can foster protective buffers against toxic stressors, such as acute socioeconomic and behavioral health needs, will remain.

**Table 3:** Emergent themes and exemplar quotes of the implementation of HUGS.

**Themes and Subthemes**	**Exemplar Quotes**
***Appropriateness***	
HUGS appropriately reaches pregnant women and families with young children who have unmet social and behavioral health needs	Most of the folks have been impacted by the pandemic. There's been a lot of different losses, [ranging] from loss in families, loss of jobs, loss of financial income…[HUGS] has been helpful and useful. I feel like short-term works. They might be referred back later on, but as a quick intervention…it is beneficial.
HUGS provides the appropriate short-term supports, including patient navigation to connect patients to community resources, and behavioral health to provide time-sensitive relief	
HUGS is an accessible and equitable program by offering phone and video visits, services in English and Spanish, and not billing health insurance, but these features are not without known drawbacks and barriers	
***Acceptability***	
The services provided and connections formed through HUGS have led to positive impacts on patients' lives	HUGS is able to tie this all in a bow, put everything together so that people can talk to each other and deliver the best care for the patients.
HUGS implements effective collaborations among providers (e.g., PCP, community health workers, social workers) and streamlines connections to care	
HUGS provides immediate access to behavioral health supports thereby reducing the time for patients to receive care and the burden on the system	
***Fidelity and Adaptations***	
The core components of HUGS have remained the same and only minor modifications have been made	Overall, everything has stayed the same: …the gift cards, the books, the community health worker involvement.
Flexibility within the program is important because patients have differing needs	
***Penetration***	
HUGS has brought together multiple hospital departments to develop new resources to better serve the patient population	Another strength of [HUGS] is that, in medicine … we work in siloes a lot. This forced a deliberate communication with each other …That's one of the strengths of this interdisciplinary collaboration that was very deliberate and eye opening.
HUGS has joined existing coalitions and has formed relationships with community organizations and should continue forging these relationships	
Referrals to HUGS are dependent on providers and their knowledge of the program, talking to families about the program, and other competing demands during the visit	
***Sustainability***	
HUGS was initially developed as a COVID-19 program to provide time-sensitive relief, but the program should be sustained as socioeconomic and health challenges will remain	I think it has potential to continue because a lot of families are benefitting from it.
Developing a plan to financially sustain HUGS is important to maintain the program	

## Discussion

Several key findings emerged when assessing HUGS/Abrazos program using the RE-AIM framework. First, patients referred to HUGS/Abrazos were representative – racially, ethnically, and socioeconomically – of the communities that were disproportionately affected by the COVID-19 pandemic, demonstrating that we are effectively reaching families in need of support. Second, there were high participation rates by referred families throughout the program, illustrating that HUGS/Abrazos filled a crucial need for patient navigation of resources and acute behavioral health support. Third, the adoption and implementation of the key components in HUGS/Abrazos were appropriate and acceptable, and they remained faithful to its original design. Finally, there is overwhelming stakeholder support in maintaining HUGS/Abrazos beyond the COVID-19 pandemic, as it has proved to be an effective delivery model in mitigating acute exacerbations of toxic stress. Taken together, these results suggest that the implementation of HUGS/Abrazos was effective in providing emergency relief to help decrease the burden on the health system.

Short-term behavioral health interventions have been shown to be effective in mitigating mental health needs during the perinatal period and among adolescents (40–44). More recently, the availability of telepsychiatry in the ED setting de-escalated mental health crises and limited the burden on an overwhelmed system during the COVID-19 pandemic (45). HUGS/Abrazos utilized similar principles in an emergency relief program to effectively address behavioral health concerns escalated by the pandemic, which were previously not addressed due to limited resources in the mental healthcare system. In terms of our patient population, HUGS/Abrazos served an age group that encompassed the perinatal period to early childhood, which is a particularly sensitive period to external adversity (5), yet few behavioral health programs address. Additionally, HUGS/Abrazos relied on an integrated structure that combined behavioral health support, patient navigation services, and direct relief. Patient navigation has previously been proven to be an effective strategy in addressing the socioeconomic factors underlying complex health needs (46–48). We combined patient navigation strategies with with resource platforms, Aunt Bertha and IRIS, to enhance centralization of resources and closed loop communication among all involve parties.

Several factors contributed to successful reach, adoption, and implementation of the HUGS/Abrazos program. Little is known about factors that support program uptake. By using the RE-AIM framework, we can begin to elucidate these factors and thereby improve the sustainability and diffusion of this innovative program and provide a roadmap for other public health innovations (49, 50). Based on a scoping review that examined factors that influenced implementation, we identified several factors that supported the implementation of HUGS/ Abrazos (49). Essential to this program was early stakeholder input from a multidisciplinary team including CHWs, the behavioral health team, and cross-departmental providers on the specific operational processes in HUGS/Abrazos allowing the program to be efficiently implemented across multiple MGH-affiliated sites. This led to the development of strong relationships within the HUGS/ Abrazos team, as well as partnering departments and organizations. We anticipate the stakeholder engagement and relationships will be critical in the maintenance of this program as has been demonstrated in the literature (51). During the inception of HUGS/ Abrazos, the team had a clear understanding of who the target population was which allowed for focused development and implementation (52). Additionally, effective recruitment of CHWs who were already familiar with the community resources and our targeted patient population ensured readiness to deliver patient navigation services. HUGS/ Abrazos program also had adequate resources to provide the necessary services. The resources included financial, personnel, and dedicated time and were a result of external funding, prioritization from the hospital system reflective of the importance of this program, and the strong relationships developed.

The strengths and limitations of program design has been discussed in detail in a prior publication (27). For our evaluation process, the use of the RE-AIM framework is a key strength that allows us to broadly assess implementation and identify areas for adaptation without disrupting intervention delivery and plan for maintenance and dissemination. One limitation is the scope of data collection, limited to EHR, administrative data, and qualitative data from those delivering the program. We used a pragmatic approach to evaluation to reduce burden and therefore we do not have data from referring providers or from families who were served by the program. As a result, we are unable to make conclusions on direct effectiveness, such as improvement in mental health or alleviation of socioeconomic needs after program participation. Additionally, there remains a perception of HUGS/Abrazos as a pandemic-specific relief program. However, the socioeconomic and behavioral health needs that HUGS/Abrazos address will outlive the pandemic, thus ensuring the program's post-COVID applicability.

In conclusion, the HUGS/Abrazos program is an emergency relief program that provides patient navigation of resources and acute behavioral health services to support vulnerable patient populations while reducing burden on an overwhelmed health system. HUGS/Abrazos serves as a protective buffer for vulnerable pregnant women and families with young children against toxic stressors exacerbated by the COVID-19 pandemic while also fostering resilience. Our evaluation of this quality improvement program, guided by the RE-AIM Framework, demonstrates that HUGS/ Abrazos was successfully adopted, reached its intended population, was effective in sustaining high participation rates and providing needed services, was acceptable, and maintained high fidelity. Next steps should focus on objective assessments of program efficacy, such as usage of validated mental health assessment instruments. Integration of social and behavioral health supports, multidisciplinary collaboration, and use of innovative tools that streamlined workflow are the basic principles that empowered the rapid implementation and effectiveness of HUGS/Abrazos, making the program an exemplary delivery model for future similar programs.

## Data Availability Statement

The raw data supporting the conclusions of this article will be made available by the authors, without undue reservation.

## Ethics Statement

Ethical review and approval was not required for the study on human participants in accordance with the local legislation and institutional requirements. Written informed consent from the participants' legal guardian/next of kin was not required to participate in this study in accordance with the national legislation and the institutional requirements.

## Author Contributions

MLi, MSi, MPe, and ET contributed to the conceptualization and design of the implementation evaluation process. MLu performed the quantitative statistical analysis and assisted with the interpretation of the data. MLi, MSi, MPe, SP, VC, and S-YTC conducted the qualitative analysis and/or assisted with the interpretation of the data. MSe, AE, CT, GK, AB, MH, MPa, WL, and ET assisted with interpretation of all data and critically reviewed the manuscript for important intellectual content. MLi and MSi drafted the first version of the manuscript. All authors contributed to manuscript revision, read, and approved the submitted version.

## Funding

Program funding was provided by the Boston Foundation to the Kraft Center for Community Health at MGH and through support by Grant no. R01HD100022 from Eunice Kennedy Shriver National Institute of Child health and Human Development awarded to Andrea G. Edlow. The content is solely the responsibility of the authors and does not necessarily represent the official views of the funders. The sponsors had no role in the evaluation, collection, analysis, and interpretation of data, writing of report, or decision to submit for publication.

## Conflict of Interest

The authors declare that the research was conducted in the absence of any commercial or financial relationships that could be construed as a potential conflict of interest.

## Publisher's Note

All claims expressed in this article are solely those of the authors and do not necessarily represent those of their affiliated organizations, or those of the publisher, the editors and the reviewers. Any product that may be evaluated in this article, or claim that may be made by its manufacturer, is not guaranteed or endorsed by the publisher.
