# Supplementary material for: Implementation Evaluation of HUGS/Abrazos During the COVID-19 Pandemic: A Program to Foster Resiliency in Pregnancy and Early Childhood
Source: Front Public Health. 2022 May 20;10:862388. doi: 10.3389/fpubh.2022.862388 (PMC9163339; doi:10.3389/fpubh.2022.862388)
Supplement: Supplementary file 1 [file Table_1.DOCX]

**Supplementary Material Table 1. Types of resource referrals made by community health workers during patient navigation**

| **Total number of families who completed at least one CHW touchpoint AND received a referral to resources** | **266** |
| --- | --- |
| **Average number of resources each family was referred to** | **mean (SD)=4.4 (3.4), range= (1, 38)** |
| **Average number of reasons for resource referrals^1^** | **mean (SD)=3.7( 2.0),  range = (1, 11)** |
| **Reasons of resource referral** | N (%) |
| Infant supplies | 160 (60.2) |
| Food | 139 (52.3) |
| Legal/housing/rent | 129 (48.5) |
| Utilities | 107 (40.2) |
| Financial assistance | 101 (38.0) |
| Clothing | 62 (23.3) |
| Childcare | 60 (22.6) |
| Child development | 49 (18.4) |
| School/education support | 26 (9.8) |
| Basic needs | 25 (9.4) |
| SNAP enrollment/assistance | 24 (9.0) |
| Family support | 19 (7.1) |
| Employment/career support | 14 (5.3) |
| Assistance related to health insurance/ medical needs | 13 (4.9) |
| Transportation | 12 (4.5) |
| Other (examples: IPV) | 28 (10.5) |

^1^A family may be provided with several resources for the same referral reason. Only the unique referral reason for each family is shown.
